# Books Reviewed

**Published:** 1867-10

**Authors:** 


### Books Reviewed.

Clinical Lectures on the Principles and Practice of Medicine. By John Hughes Bennett, M. D., etc., etc.

This work, on the practice of medicine, has been for a long time out of print, and this fourth edition will be received by the profession with much satisfaction; it has long been a standard work of acknowledged merit, and without change in the text would have been reeeived with favor.

This edition has reoeived many additions and some portions are entirely new; sections upon molecular and cell theories of organization, general laws of nutrition and innervation, of inflammation and tuberculosis, have been re-written. In the treatment of morbid growths the author has introduced a note from *M.* Velpeau, in which that surgeon demonstrated the correctness of an opinion long held by the author, that *true cancer* may be permanently extirpated with the knife. He says: "The facts Velpeau has recorded ought to put an end to further discussion on the subject."

The author has introduced a full discussion of the *natural progress of disease; the importance of pathology and improved diagnosis; the fallacy of the change of*

*type theory; and an inquiry into the present means of treatment, advocating the id ea that physiology and pathology constitute the true foundations of medical practice.*

T he work is complete, embraciug a wide range of topics and arranged with special reference to the wants of the general practitioner. It is illustrated with five hundred and thirty-seven wood cuts, which add to its descriptions of disease, and of pathological changes a clearness of impression which could not otherwise be obtained. On this account the book has attractions for the student of medicine unequaled by any other. It is unnecessary to add that in its present edition it comprises what is known of disease, and of its rational modes of cure; and is a safe and satisfactory guide not ouly in pathology but also in treatment.

---

The Physiology and Pathology of the Mind. By Henry Maudsley, M. D., London.

The author has treated in this work, mental phenomena physically rather than metaphysically, and brought the instrnctive instances presented by the unsound mind, to aid in the interpretation and explanation of the obscure questions in mental science. He says: "it has been my desire to do what I could in order to put a happy end to the 'inauspicious divorce' between the physiology and pathology of mind, and to effect a reconciliation between these two branches of the same science."

It is impossible to go into any satisfactory analysis of this work, and inconsistant with our space to give even detailed account of its range and scope. The whole physiology of mental operations, the causes of perversion, the various forms of deviation and the import of these abnormal manifestations are discussed in a philosophical and scholarly manner, and cannot fail to interest and instruct every reader. It is a *new book*, the whole of which is so rational and logical as to be worthy careful perusal.

Speaking physiologically and anatomically of the brain he says: "It is extremely probable that different convolutions of the brain do subserve different functions in mental life; but the precise mapping out of the cerebral surface, and the classification of the mental faculties, which the phrenologists have rashly made, will not bear scientific examination. That the broad and prominent forehead indicates great intellectual power, was believed in Greece; and is commonly accepted as true now; the examination of the brains of animals and idiots, and the comparison of the brain of the lowest savage with the brain of the civilized European certainly tend to strengthen the belief. Narrow and pointed hemispheres assuredly do mark an approach to the character of the monkey's brain. There is some reason to believe also, that the upper part of the brain and the posterior lobes have more to do with feeling than with the understanding. Huschkle has found these parts to be proportionably more developed in women than in men; and Schrœder Van der Kolk thought that his pathological researches had afforded him the most convincing proofs that the anterior lobes of the brain were the seat of the higher intellectual faculties, while the upper and posterior lobes ministered rather to the emotional life. Recently some observations have been made with he view of establishing a theory that a portion of the anterior lobe. the third frontal convolution of the left hemisphere, was the seat of langua ge; but the observations reported are unsatisfactory, directly contradictory observations are overlooked, and it is contrary to the first principles of psychology to suppose that language, complex and organic as it is in its intellectual character as the sign or symbol of the idea, can have so limited and defined a seat in the brain, On the whole, it must be confessed, so far, we have not any certain and definite knowledge of the functions of the different parts of the cerebral convolutions. The anatomists cannot even agree on any convolution as peculiar to man; all that they can surely say is, that his convolutions are more complex and less symmetrical than those of the monkey. 'If man was made in the image of God, he was also made in the image of an ape.'" This last conclusion the author quotes from Hallam, in his introduction to the *History of Europe.* We have indulged in too lengthy extracts, and close our brief notice of this book with, an invitation to all, to read it carefully for themselves.

Published by D. Appleton & Co., New York.

---

The Medical Use of Electricity, with illustrative cases. By Geo. M. Beard, M. D. and A. D. Rockwell, M. D. New York: Wm. Wood & Co., 1867.

The therapeutical value, as a tonic, of general electerization in the various neurotic affections is the especial theme of this monograph; the authors presenting the results of their united experiments and experience in a most satisfactory manner. As a body, physicians have manifested great apathy towards electro-therapeutics, arising from the unsatisfaciory results usually obtained by them, but which the authors attribute to the imperfect employment of this agent. The treatise is entirely freed from the usual ambiguity aud mystification of terms found in works upon this topic, simplicity and clearness of language being maintained throughout.

---

A Practical Guide to the Study of the Diseases of the Eye; their Medical and Surgical Treatment. By Henry W. Williams, M. D., etc,, etc.

It is but few months since we had occasion to speak of the merits of the first edition of this work; and we can now add but little to our former expressions of approval. The book contains the essential and practical science of ophthalmology as now understood and practiced, and to it as an appendix has been added the author's Prize Essay upon Recent Advances in Ophtalmic Science, thus presenting concisely and clearly, the new facts and principles regarding the optical powers and functions of the eye, which have recently been discovered and demonstrated by Donders, Græfe, Helmboltz and others, and which has done so much to give ophthalmology a high place as one of the departments of medical science· The author has not attempted to embody all that is known upon the subjects considered, but has adapted his book to the wants of the student and general practitioner. It contains, in condensed form, most of what is really practical in this department of medicine, and shows in every chapter a thorough, practical, as well as theoretical acquaintance with the topics discussed, so that the work may be regarded as, not only recent in theory, but well considered and truthful in its practical conclusions.

The great need of more careful attention to the diseases of the eye, cannot have escaped the attention of any who are at all familiar with the condition of the profession in this respect. This book will supply all that can be desired by the student and general practitioner, and its value cannot be over-estimated.

---

Essentials of the Principles and Practice of Medicine; a handy book for Students and Practitioners. By Henry Hartshorne, M. D., Professor of Hygiene in the University of Pennsylvania, etc., etc. Philadelphia: Henry C. Lea, 1867.

For many years Dr. Hartshorne has been constantly engaged as a teacher of medicine, and the extensive experience during a period of twenty years, of both private and hospital practice, renders his opinions of great value. The author has not aimed at an elaborate treatise on the principles and practice of medicine, but simply to present his experience in the most concise and practical form, in which endeavor he has been eminently successful. In the treatment of disease such remedies are enumerated as the author has found from personal experience to be the most reliable. This work will prove a valuable companion to both student and practitioner.

---

Brand and Taylor's Chemistry. Second Edition. Philadelphia: Henry C. Lea, 1867.

The author states the object in undertaking this work, was to furnish the reader, whether a medical student or a man of the world, with a plain introduction to the science and practice of chemistry.

The author adheres to the old method of notation, based upon the equivalent or combining weights of bodies, which, though not perfect, he says, "is based upon simple and intelligible principles." Speaking of the new methods of notation, he says, "they must be regarded as still upon their trial. Gerhardt's system, which a few years ago was generally adopted by 'advanced' chemists, has now given place to another system, and the extinction of this is threatened by a third and an entirely different system, recently proposed by Sir Benjamin Brodie, Professor of Chemistry in the University of Oxford." Again, he says, "it will be found that in the best modern works on chemistry, in the English and French languages, the ordinary notation is adopted, and the new notation ignored even by writers, who have been or are advocates of a change."

This work on chemistry we believe is very popular, both with students and chemists, and though we have not personal knowledge to judge of its merits, and could not, even if we had space, review its ground and speak in detail of its worth, still we see by the little we know, how much might be understood by careful study of this author, and believe his work highly adapted to the wants of students and all others who desire knowledge of the science of chemistry in its present advanced state.

